# Eight-Centimeter Gallbladder Stone Post-Roux-en-Y Gastric Bypass: A Case Report

**DOI:** 10.7759/cureus.35604

**Published:** 2023-02-28

**Authors:** Lauren Hughes, Maryam Morris, Mohamed Hegazy, Fremita Fredrick, Frederick Tiesenga, Juaquito Jorge

**Affiliations:** 1 Medicine, Saint James School of Medicine - Anguilla Campus, The Quarter, AIA; 2 General Surgery, West Suburban Medical Center, Oak Park, USA; 3 Surgery, Community Medical Center, Chicago, USA; 4 Medicine, Saint James School of Medicine - St Vincent Campus, Arnos Vale, VCT; 5 Surgery, Avalon University School of Medicine, Ohio, USA; 6 General and Bariatric Surgery, West Suburban Hospital, Oak Park, USA

**Keywords:** giant gallbladder stone, s: bariatric, bariatric surgery/therapeutic use, bariatric medicine, roux-en-y gastric bypass (rygb), cholelithiasis, symptomatic cholelithiasis

## Abstract

Cholelithiasis occurs when a stone forms in the gallbladder; when symptoms develop, the condition is termed symptomatic cholelithiasis. The correlation between bariatric surgery and post-operative symptomatic cholelithiasis has long been established. Presented is a case of a 56-year-old female status post-Roux-en-Y gastric bypass who developed symptomatic cholelithiasis and subsequently underwent cholecystectomy with the removal of an 8-centimeter (cm) gallbladder stone. This case report explores the benefits and limitations of watchful waiting versus prophylactic concomitant cholecystectomy among bariatric surgery patients, noting the difference between the bariatric sleeve and bypass anatomy for managing biliary complications.

## Introduction

Cholelithiasis is a condition describing when a stone forms in the gallbladder. The two primary types of gallstones are cholesterol and pigment stones. Cholesterol stones are the predominant type in the United States and Western Europe, comprising 90% of cases. In the United States, approximately 20 million people have cholelithiasis; however, 70% to 80% do not experience any symptoms and are therefore classified as having asymptomatic cholelithiasis [1]. Yearly, 4% of those with asymptomatic cholelithiasis develop symptoms and are then classified or diagnosed with symptomatic cholelithiasis. Symptoms of cholelithiasis include excruciating pain in the right upper quadrant or epigastrium, typically following a fatty meal. The pain may also radiate to the back and right shoulder. Symptomatic cholelithiasis is an indication of cholecystectomy, a surgical procedure in which the gallbladder is removed, typically performed laparoscopically [1].

The pathogenesis of cholesterol stones involves gallbladder hypomotility, bile supersaturation with cholesterol, accelerated nucleation of cholesterol crystals, and mucus hypersecretion within the gallbladder, resulting in the trapping of nucleated crystals [2]. Risk factors precipitating stone formation include age, sex, oral contraceptive pill (OCP) use, obesity, rapid weight loss, and gallbladder stasis [2]. Bariatric surgery and the resultant, often rapid, weight loss has been linked to symptomatic cholelithiasis and is of particular interest.

Bariatric surgeries available to patients include laparoscopic adjustable gastric banding, laparoscopic sleeve gastrectomy, Roux-en-Y gastric bypass (RYGB), and duodenal switch [3]. Research has shown that the likelihood of developing symptomatic cholelithiasis is increased with each procedure. However, there are varying degrees in the extent each procedure increases the likelihood of developing symptomatic cholelithiasis. For example, RYGB had a 6% to 50% incidence compared to approximately 1% to 8% in laparoscopic sleeve gastrectomy and roughly 4% in laparoscopic gastric banding [4].

## Case presentation

A 56-year-old female with a past medical history significant for morbid obesity status post gastric bypass with concomitant weight loss, chronic obstructive pulmonary disease, hypertension, diabetes mellitus, and obstructive sleep apnea presented to the outpatient surgery office with complaints of abdominal bloating, gas, heartburn/dyspepsia, and epigastric pain. The patient initially complained of intermittent abdominal bloating and gas but then reported heartburn/dyspepsia and epigastric pain, specifically postprandial right upper quadrant (RUQ) pain. The patient denied chest pain, shortness of breath, or difficulty breathing. The patient’s surgical history was only positive for RYGB undergone two years prior. The patient was able to essentially resolve DM with a most recent hemoglobin A1C level of 5.9% and showed significant improvement in COPD, hypertension, and OSA conditions and symptoms; however, these conditions were not entirely resolved and still required medication and treatment regimens after two years of RYGB. At the time of the initial consultation for bariatric surgery, the patient completed a standardized gallbladder screening questionnaire (Appendix Table 1). The patient had one positive result: "Yes,” to “Do you have diarrhea after eating a high-fat meal, milk, or dairy products?”; however, the rest of the screening questionnaire results were negative. Patient-reported or radiographically confirmed gallstone was not present in the patient before RYGB bariatric surgery. The patient had no known allergies, and their past medical history was otherwise non-contributory.

**Figure 1:**
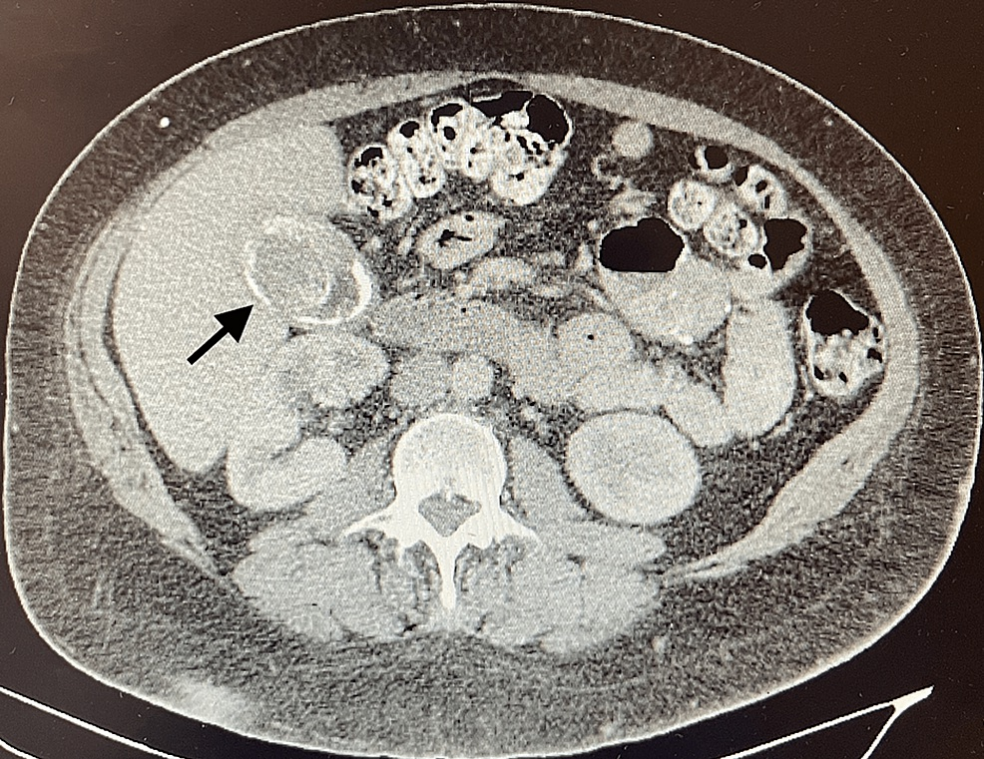
CT of the abdomen and pelvis with intravenous contrast showing large, partially calcified gallstones, essentially filling the entire gallbladder lumen

The patient's pre-RYGB body mass index (BMI) was 63.3, and their BMI at the time of the current presentation was 39.5. The patient’s abdomen was soft on physical examination, with a large overhanging pannus. There was no tenderness, guarding, or rebound tenderness. The patient did not have any scleral icterus or jaundice. A computed tomography (CT) scan of the abdomen and pelvis with intravenous contrast showed large gallstones, essentially filling the entire gallbladder lumen, with no specific features of acute inflammation (Figure 1). The patient also completed an esophagogastroduodenoscopy (EGD) for heartburn, which showed normal mucosa in the whole esophagus, evidence of a previous gastro-jejunal bypass surgery was seen in the gastric-jejunal anastomosis, a small esophageal hiatal hernia, and a healthy appearing roux limb. With increasingly symptomatic cholelithiasis confirmed radiographically recommendation was made for cholecystectomy. The patient’s informed witnessed consent, and pre-operative clearance was obtained.

The patient underwent robotic-assisted cholecystectomy without complications. Robotic-assisted laparoscopic cholecystectomy was performed based on surgeon preference and related decreased surgical invasiveness and post-operative infection rate. During the surgical procedure, the gallbladder was noted to be significantly distended and had an extremely large gallstone impacted in the neck. In addition, the patient had a large fatty stiff liver, which made retraction and exposure extremely difficult. The gallbladder was removed from the liver using electrocautery, placed in a retrieval bag, and sent to pathology for analysis. Of note, because of the large, impacted stone, the left paramedian incision trocar site had to be extended to get the gallbladder and stone out, and the fascia closed afterward. 

**Figure 2:**
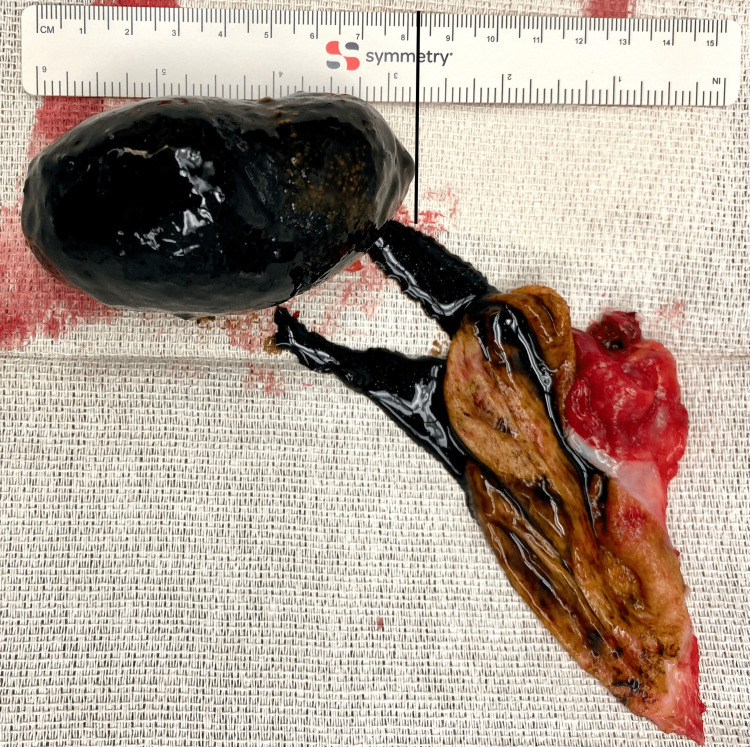
Resected opened gallbladder with an 8 cm by 5 cm by 5 cm oblong green stone with ulcerated mucosa

Pathology of the resected gallbladder identified an opened gallbladder with an 8 cm by 5 cm by 5 cm oblong green stone with ulcerated mucosa (Figure 2). No masses or defects were identified in the thickest portion of the gallbladder wall, which measured 0.2 cm.

Post-operatively the patient did initially experience some difficulty with pain control after receiving a transversus abdominis plane (TAP) block, a 2 milligram (mg) dose of hydromorphone, and a 1 mg dose of Acetaminophen. The patient then had an uncomplicated course and was discharged home on the same day per protocol. At the patient’s first postoperative visit, they reported that their abdominal bloating had improved; however, they were experiencing RUQ and midback pain radiating to the lower back and abdominal area. At the patient’s next postoperative visit, they reported complete resolution of their pain and cholelithiasis symptoms.

## Discussion

Although there have been several treatments and prophylactic management strategies for cholelithiasis in status post-bariatric surgery patients, there is no ​​standardized approach. This case report presents a patient that suffered from a giant gallbladder stone measuring 8 cm, post-RYGB surgery. Gallstones measuring more than 5 cm are rare, and only a few reported cases in the literature. The size of the gallstone is a significant risk factor since gallstones larger than 3 cm have a higher rate of complications, including increased risk for gallbladder cancer, biliary enteric fistula, gallstone ileus, and higher technical difficulties during laparoscopic cholecystectomy [5]. This patient with an 8 cm gallstone is a rare but vital case to emphasize the importance of screening and prophylactic treatment approaches available to reduce gallstone complications in post-bariatric surgery patients.

Two main treatment and prophylactic management strategies for cholelithiasis in status post-bariatric surgery patients are watchful waiting and prophylactic concomitant cholecystectomy. In watchful waiting, the physician monitors the patient for symptomatic cholelithiasis and the development of symptomatic cholelithiasis and only performs cholecystectomy at that time if indicated. This approach's advantages include avoiding a more invasive, possibly unnecessary operation. However, disadvantages include delays in treating cholelithiasis, perhaps leading to more significant biliary complications, such as biliary colic, cholecystitis, cholangitis, choledocholithiasis, pancreatitis, obstructive cholestasis, fistula, perforation, and empyema as was seen in this case with the patient’s development of biliary colic [2,4,6,7].

In prophylactic concomitant cholecystectomy, the surgeon performs a prophylactic concomitant cholecystectomy during bariatric surgery. The advantages of this approach are that there is no risk for future cholelithiasis development and resulting procedures. During the early 2010s, many surgeons performed routine prophylactic concomitant cholecystectomies in all bariatric surgery patients. A study from 2022 concluded that prophylactic concomitant cholecystectomies could be more challenging to complete due to suboptimal trocar placement and high visceral obesity. Routine prophylactic concomitant cholecystectomies should not be performed in all bariatric patients as they have increased operation time. Many morbidly obese patients have comorbidities such as hypertension, diabetes, and microcirculation disorders which place them at risk for complications related to prolonged operation time [7]. Another study found that routine prophylactic concomitant cholecystectomies performed in all bariatric patients resulted in an additional 744 cholecystectomies, of which their data did not indicate a benefit to offset the increased costs and risks [8]. Thus, routine prophylactic concomitant cholecystectomies in all bariatric surgery patients are no longer recommended, given the lack of solid, beneficial evidence and increased unnecessary procedures with possible complications [4,9,10]. Elective/Selective prophylactic concomitant cholecystectomy is another approach in which the surgeon performs a prophylactic concomitant cholecystectomy only on patients with pre-operative cholelithiasis; however, experts seem divided on this surgical approach. One study demonstrated that this method could significantly increase surgical time, hospital stay, and morbidity [11]. Another study suggested no difference in hospitalization length between patients undergoing simultaneous cholecystectomy and those who had bariatric surgery alone, with the only significant difference being the duration of the operation [12]. The research suggests elective/selective cholecystectomy may benefit some patients with moderate to high risk of symptomatic cholelithiasis [6-8,11,12].

The Roux-En-Y procedure causes rapid weight loss and a higher total weight loss percentage leading to a higher risk of symptomatic cholelithiasis than in patients who undergo sleeve gastrectomy and gastric banding. There is a strong correlation between initial weight loss, losing 1.5 kilograms (kg) per week, and higher excess BMI loss percentage and symptomatic cholelithiasis [13]. Additionally, gastric bypass interferes with biliary contraction mechanisms and enterohepatic circulation, causing a higher risk of biliary complications. Furthermore, the resulting anatomy of gastric bypass makes access for endoscopic retrograde cholangiopancreatography (ERCP) limited or would require specialized approaches such as lap-assisted ERCP, ERCP performed via colonoscope or balloon-assisted enteroscopy. TThus, patients are at higher risk of severe biliary complications and must be managed accordingly with careful monitoring [4,9].

The data surrounding risk versus benefits for prophylactic concomitant cholecystectomy for patients with moderate to high risk for symptomatic cholelithiasis and its related complications has found that elective/selective prophylactic concomitant cholecystectomy should be strongly considered and may be necessary for these patients [6,11,12]. This case report proposes a standardized approach in which a physician uses their clinical reasoning to determine a patient’s individualized overall risk for developing symptomatic cholelithiasis and its related complications after bariatric surgery by considering: the patient’s results from a gallbladder screening questionnaire; the risk associated with the patient’s specific bariatric procedure; and other patient-specific risk factors (gender, age, etc.). The physician should then use the individualized overall risk to determine whether prophylactic concomitant cholecystectomy during bariatric surgery is beneficial. Screening for symptomatic cholelithiasis before bariatric surgery using a questionnaire such as the self-report Biliary Symptoms Questionnaire (BSQ), the shortened version (sBSQ), The Otago gallstones condition-specific questionnaire (CSQ), and others has been proven reliable, as patients who experience gallbladder symptoms are at an increased risk for developing symptomatic cholelithiasis and its related biliary complications [8,14-17].

The patient in the case report answered “Yes,” to one of the gallbladder screening questionnaire questions utilized by the particular office, indicating a low but positive screen. The patient then underwent watchful waiting with monthly follow-up visits at the outpatient surgery office. However, cholelithiasis was only detected when it became symptomatic. If the proposed standardized approach were utilized for the presented case, the patient’s low but positive gallbladder questionnaire screen, the increased risk associated with the Roux-En-Y procedure, and the presence of patient-specific risk factors (gender) would have all been considered and through clinical reasoning the patient would have determined to be at moderate overall risk. Subsequently, the patient would have undergone elective/selective prophylactic concomitant cholecystectomy and never subjugated to the increased pain and more difficult and invasive surgery due to a giant 8 cm gallbladder stone.

## Conclusions

The presented case is a 56-year-old female status post-RYGB who suffered from symptomatic cholelithiasis due to an 8 cm giant gallbladder stone. This case highlights that current studies do not present a ​​standardized approach for the treatment and prophylactic management strategies for cholelithiasis in status post-bariatric surgery patients and a need for a more efficient and standard approach. While the research does not recommend routine prophylactic concomitant cholecystectomy on all bariatric surgery patients, evidence supports that elective/selective prophylactic concomitant cholecystectomy benefits patients with moderate to high risk of symptomatic cholelithiasis and related complications. This case report proposes a standardized approach in which a physician uses their clinical reasoning to determine a patient’s individualized overall risk for developing symptomatic cholelithiasis and its related complications after bariatric surgery by considering: the patient’s results from a gallbladder screening questionnaire; the risk associated with the patient’s specific bariatric procedure; and other patient-specific risk factors. Finally, elective/selective prophylactic concomitant cholecystectomy should be performed in those of moderate to high risk.

## Appendices

**Table 1:** Gall bladder screening

	Yes	No
Do you have abdominal pains on and off after a high fat meal?		
Do you have sharp pain to your right side after eating?		
Do you feel nauseous after eating?		
Do you experience bloating after eating?		
Do you constantly burp or belch?		
Do you have diarrhea after eating a high fat meal, milk or dairy products?		
